# Correspondence: Risk perception of COVID-19 and vaccine uptake among patients with chronic illnesses at a tertiary health facility in Nigeria

**DOI:** 10.4314/gmj.v59i1.8

**Published:** 2025-03

**Authors:** Hinpetch Daungsupawong, Viroj Wiwanitkit

**Affiliations:** Phonhong, Lao People's Democratic Republic; Department of Research Analytics, Saveetha Dental College and Hospitals, Saveetha Institute of Medical and Technical Sciences Saveetha University, Chennai, India

Dear Editor,

This is a response to published research on “The risk perception of COVID-19 and vaccine uptake among patients with chronic illnesses at a tertiary health facility in Nigeria.^1^” Studies on the risk perception of COVID-19 vaccination among chronically ill patients in tertiary care centres provide useful information. However, several methodological issues have emerged, limiting its effectiveness, particularly in the Nigerian context. This is especially true for how risk perception affects vaccination uptake. Can the authors explain how they expect to demonstrate causation in future studies? Longitudinal designs may be more suited to tracking changes in attitudes and behaviours over time, particularly if new information on COVID-19 and its vaccines becomes available.

The sampling methods employed include basic random sampling and proportional distribution. However, the inclusion criteria for this study raise concerns regarding possible bias. How does this group, who also suffers from chronic conditions, influence general awareness of vaccination and risk perception?

The findings show that COVID-19 vaccinations are being taken up. More than two-thirds of respondents have had vaccination experience; however, vaccine hesitation is attributed to a fear of the unknown and concern about adverse effects. More thorough qualitative data could help researchers better understand immunisation hurdles. Furthermore, the discovery that marital status increases the chance of immunisation is intriguing. or inhibits vaccination.

Future research may examine the effect of community engagement and public health campaigns on vaccine acceptability as new virus types and vaccines are introduced. It will be critical to monitor changes in risk perception and vaccination uptake over time. By considering these shift factors, public health efforts may be better targeted to patients' needs and concerns. This will eventually lead to higher vaccination rates and better health outcomes for these vulnerable populations.

## Authors' Response

We are thankful for the valuable feedback on our work. We admit the limitations mentioned and would like to clarify the following:

**Causality and Study Design:** We agree that the study utilised a cross-sectional design, which inherently limits the ability to establish causal relationships between risk perception and vaccine uptake. Our objective was to explore associations rather than determine causality. We agree that a longitudinal study would provide a more robust approach to track changes in attitudes and behaviours over time. Further longitudinal studies should be set up to assess the changes in risk perception and vaccine uptake as information about COVID-19 and vaccines advances.

**Inclusion Criteria and Sampling Methodology:** This study employed simple random sampling and proportional allocation to select participants from outpatient clinics, which was appropriate for a tertiary care setting to ensure a good representation of patients with chronic illnesses. However, we recognise the concern about potential bias influencing general awareness of vaccination and risk perception. Future studies could broaden the inclusion criteria to capture a more diverse population and consider stratified sampling to enhance representativeness.

**Qualitative enrichment:** We acknowledge the suggestion for more comprehensive qualitative data to deepen our understanding of immunisation barriers. This would provide a richer perspective on vaccine hesitancy, particularly in exploring the nuanced reasons behind fear of the unknown and concerns about adverse effects. Future studies could include in-depth interviews and focus group discussions to complement our study's findings.

**Marital Status and Vaccination Uptake:** These research studies found a statistically significant relationship between marital status and vaccine uptake. This finding is quite interesting and calls for further research. Future studies could explore the underlying cultural, social, or support system factors influencing this association.

**Future Research Directions:** We agree that further studies should be conducted to ascertain how community engagement and public health campaigns can promote vaccine acceptability. In addition, as the public health scene for COVID-19 continues shifting because of emerging variants and the innovations of new vaccines, it will equally be essential to measure how the perception of risk and uptake will change with time. Future studies could also evaluate what role different channels of information dissemination play in ameliorating vaccine hesitancy.

**Conclusion:** We appreciate the constructive feedback and the opportunity to clarify the scope and limitations of our study. Our research was not aimed at establishing causality but rather at exploring associations between risk perception and vaccine uptake. We acknowledge the need for longitudinal and qualitative studies to build upon our findings and provide a more comprehensive understanding of vaccination behaviours among patients with chronic illnesses.

We appreciate everyone who has engaged in our work and look forward to contributing more knowledge on COVID-19 risk perception and vaccine uptake.
